# Supplementary material for: Frequency modulation on magnons in synthetic dimensions
Source: Nat Commun. 2025 Apr 9;16:3356. doi: 10.1038/s41467-025-58582-z (PMC11982341; doi:10.1038/s41467-025-58582-z)
Supplement: Supplementary file 1 — Supplementary Information [file 41467_2025_58582_MOESM1_ESM.pdf]

Supplementary Information for: Frequency modulation on magnons in synthetic dimensions

Meng Xu,† Chensong Hua,‡ Yan Chen,*,†,¶ and Weichao Yu*,‡,¶,§

†*Department of Physics, Fudan University, Shanghai 200433, China*

‡*Institute for Nanoelectronic Devices and Quantum Computing, Fudan University, Shanghai 200433, China*

¶*State Key Laboratory of Surface Physics, Fudan University, Shanghai 200433, China*

§*Zhangjiang Fudan International Innovation Center, Fudan University, Shanghai 201210, China*

E-mail: yanchen99@fudan.edu.cn; wcyu@fudan.edu.cn

Derivation of the effective tight-binding model

Coupled-mode equation

We start from the Schrödinger-like equation (Eq.(3) in the main text) that

$$i\dot{m}_+ = \gamma A \nabla^2 m_+ - \gamma \left[H + H_\mathrm{m}\cos(\Omega t + \phi)\right] m_+, \tag{1}$$

and treat the "wave function" m_+ as superposition of occupation on each synthetic lattice labeled by n

$$m_+(l,t) = \sum_n C_n(t) e^{i(\omega_n t - k_n l)}. \tag{2}$$

Plugging Eq.(2) into Eq.(1) and cancelling the term $e^{ik_n l}$ on both sides, we obtain

$$i\sum_n \left(\dot{C}_n(t)e^{i\omega_n t} + i\omega_n C_n(t)e^{i\omega_n t}\right) = \left(-\gamma A k_n^2 - \gamma H\right)\sum_n C_n(t)e^{i\omega_n t} - \gamma H_{\mathrm{m}}\frac{e^{i\Omega t}+e^{-i\Omega t}}{2}\sum_n C_n(t)e^{i\omega_n t}, \tag{3}$$

where we assume $\phi = 0$ and adopt the identity relation $2\cos(\Omega t) = e^{i\Omega t} + e^{-i\Omega t}$.

According to the spin-wave dispersion $\omega_n - \gamma A k_n^2 - \gamma H = 0$ and the denotation $g = -\gamma H_{\mathrm{m}}/2$, Eq.(3) can be further simplified as

$$i\sum_n \dot{C}_n(t)e^{i\omega_n t} = g\left[\sum_n C_n(t)e^{i(\omega_n+\Omega)t} + \sum_n C_n(t)e^{i(\omega_n-\Omega)t}\right]. \tag{4}$$

We focus on the dynamics of m-th mode, and Eq.(4) can be expanded as

$$\begin{aligned} i\Big[\cdots + \dot{C}_{m-1}(t)e^{i\omega_{m-1}t} + \dot{C}_m(t)e^{i\omega_m t} + \dot{C}_{m+1}(t)e^{i\omega_{m+1}t} + \cdots\Big] = \\ g\left[\cdots + C_{m-1}(t)e^{i(\omega_{m-1}+\Omega)t} + C_m(t)e^{i(\omega_m+\Omega)t} + C_{m+1}(t)e^{i(\omega_{m+1}+\Omega)t}\cdots\right] + \\ g\left[\cdots + C_{m-1}(t)e^{i(\omega_{m-1}-\Omega)t} + C_m(t)e^{i(\omega_m-\Omega)t} + C_{m+1}(t)e^{i(\omega_{m+1}-\Omega)t}\cdots\right]. \end{aligned} \tag{5}$$

Since the m-th mode varies with the angular frequency of ω_m, we eliminate the terms on the left hand side which vary faster or slower than $e^{i\omega_m t}$, and Eq.(5) becomes

$$\begin{aligned} i\dot{C}_m(t) = g\Big[\cdots + \overbrace{C_{m-1}(t)e^{i(\omega_{m-1}-\omega_m+\Omega)t}}^{\text{leading-order term}} + C_m(t)e^{i\Omega t} + C_{m+1}(t)e^{i(\omega_{m+1}-\omega_m+\Omega)t} + \cdots\Big] + \\ g\Big[\cdots + \underbrace{C_{m+1}(t)e^{i(\omega_{m+1}-\omega_m-\Omega)t}}_{\text{leading-order term}} + C_m(t)e^{-i\Omega t} + C_{m-1}(t)e^{i(\omega_{m-1}-\omega_m-\Omega)t} + \cdots\Big]. \end{aligned} \tag{6}$$

We assume that the driving frequency of modulation Ω is close to the energy spacing between neighbour modes around mode m, i.e., $\omega_m - \omega_{m-1} < \Omega < \omega_{m+1} - \omega_m$, so that we can keep the slowest terms on the right hand side of Eq.(6). Substituting the label from a specific mode m to arbitrary modes n, we can obtain the coupled-mode equation (Eq.(5) in the main text)

$$i\dot{C}_n = g\left[e^{i(\omega_{n+1}-\omega_n-\Omega)t}C_{n+1} + e^{-i(\omega_n-\omega_{n-1}-\Omega)t}C_{n-1}\right]. \tag{7}$$

Effective Hamiltonian 1

The coupled-mode equation Eq.(7) describes the dynamics of a particle characterized by the state $|\psi(t)\rangle = \sum_n C_n(t) a_n^\dagger |0\rangle$ governed by Schrödinger equation $i\partial_t|\psi(t)\rangle = \mathcal{H}|\psi(t)\rangle$, with $a_n^\dagger$ (a_n) the creation (annihilation) operator applied on vacuum state $|0\rangle$. The effective Hamiltonian reads

$$\mathcal{H} = \sum_n g\left(a_n^\dagger a_{n+1} e^{i(\omega_{n+1}-\omega_n-\Omega)t} + a_{n+1}^\dagger a_n e^{-i(\omega_{n+1}-\omega_n-\Omega)t}\right). \tag{8}$$

We choose the operator $\mathcal{U}_1{}^\dagger(t) = \mathrm{diag}[\cdots, e^{i(\omega_{n-1}-(n-1)\Omega)t}, e^{i(\omega_n - n\Omega)t}, e^{i(\omega_{n+1}-(n+1)\Omega)t}, \cdots]$ and perform gauge transformation, so that $\tilde{a}_n^\dagger(t) = e^{-i(\omega_n - n\Omega)t} a_n^\dagger(t)$ and $\tilde{a}_n(t) = e^{i(\omega_n - n\Omega)t} a_n(t)$. The effective Hamiltonian (Eq.(7) in the main text) is obtained after gauge transformation $\tilde{\mathcal{H}} = \mathcal{U}_1(t)^\dagger \mathcal{H} \mathcal{U}_1(t) - i\mathcal{U}_1^\dagger(t)\mathrm{d}_t\mathcal{U}_1(t)$ [1], which is derived as

$$\begin{aligned}
\tilde{\mathcal{H}} =& \sum_n g\left[\tilde{a}_n^\dagger e^{i(\omega_n - n\Omega)t} \tilde{a}_{n+1} e^{-i(\omega_{n+1}-(n+1)\Omega)t} e^{i(\omega_{n+1}-\omega_n-\Omega)t}\right] \\
&+ \sum_n g\left[\tilde{a}_{n+1}^\dagger e^{i(\omega_{n+1}-(n+1)\Omega)t} \tilde{a}_n e^{-i(\omega_n - n\Omega)t} e^{-i(\omega_{n+1}-\omega_n-\Omega)t}\right] \\
&+ \sum_n \left(-i\frac{\mathrm{d}}{\mathrm{d}t}(-i(\omega_n - n\Omega)t)\right) \tilde{a}_n^\dagger e^{i(\omega_n - n\Omega)t} \tilde{a}_n e^{-i(\omega_n - n\Omega)t} \\
=& \sum_n g\left(\tilde{a}_n^\dagger \tilde{a}_{n+1} + \tilde{a}_{n+1}^\dagger \tilde{a}_n\right) + \sum_n (n\dot{\Omega}t + n\Omega - \omega_n)\tilde{a}_n^\dagger \tilde{a}_n. \quad \text{(effective Hamiltonian 1)}
\end{aligned} \tag{9}$$

Effective Hamiltonian 2

We start from Eq.(6) where have kept only leading-order terms during the derivation above. In this section, we keep more terms with wavy line as shown in Eq.(10) and we can obtain the corre-

sponding coupled-mode equation

$$\begin{aligned} i\dot{C}_m(t) =& g\Big[\cdots + \underset{\sim\sim\sim}{C_{m-1}(t)e^{i(\omega_{m-1}-\omega_m+\Omega)t}} + \underset{\sim\sim\sim}{C_m(t)e^{i\Omega t}} + \underset{\sim\sim\sim}{C_{m+1}(t)e^{i(\omega_{m+1}-\omega_m+\Omega)t}} + \cdots\Big] + \\ & g\Big[\cdots + \underset{\sim\sim\sim}{C_{m+1}(t)e^{i(\omega_{m+1}-\omega_m-\Omega)t}} + \underset{\sim\sim\sim}{C_m(t)e^{-i\Omega t}} + \underset{\sim\sim\sim}{C_{m-1}(t)e^{i(\omega_{m-1}-\omega_m-\Omega)t}} + \cdots\Big] \\ =& g(e^{i\Omega t} + e^{-i\Omega t})\left[e^{i(\omega_{m+1}-\omega_m)t}C_{m+1} + e^{-i(\omega_m-\omega_{m-1})t}C_{m-1} + C_m\right], \\ =& 2g\cos(\Omega t)\left[e^{i(\omega_{m+1}-\omega_m)t}C_{m+1} + e^{-i(\omega_m-\omega_{m-1})t}C_{m-1} + C_m\right]. \end{aligned} \tag{10}$$

Following the same notation as in Eq.(8), we obtain the effective Hamiltonian according to the coupled-mode equation Eq.(10)

$$\begin{aligned} \mathcal{H}' &= \sum_n 2g\cos(\Omega t)a_n^\dagger a_n + \sum_n 2g\cos(\Omega t)\left[a_n^\dagger a_{n+1}e^{i(\omega_{n+1}-\omega_n)t} + a_{n+1}^\dagger a_n e^{-i(\omega_{n+1}-\omega_n)t}\right] \\ &\sim \sum_n 2g\cos(\Omega t)\left[a_n^\dagger a_{n+1}e^{i(\omega_{n+1}-\omega_n)t} + a_{n+1}^\dagger a_n e^{-i(\omega_{n+1}-\omega_n)t}\right], \end{aligned} \tag{11}$$

where the first term $\sum_n 2g\cos(\Omega t)a_n^\dagger a_n$ is eliminated because of its independence on lattice site n which will not contribute to the dynamics. We transform the creation and annihilation operators as $\breve{a}_n^\dagger = e^{-i\omega_n t}a_n^\dagger$ and $\breve{a}_n = e^{i\omega_n t}a_n$ by choosing the operator $\mathcal{U}_2{}^\dagger(t) = \mathrm{diag}[\cdots, e^{i\omega_{n-1}t}, e^{i\omega_n t}, e^{i\omega_{n+1}t}, \cdots]$, and we can obtain the gauge-transformed Hamiltonian according to $\breve{\mathcal{H}} = \mathcal{U}_2(t)^\dagger\mathcal{H}'\mathcal{U}_2(t) - i\mathcal{U}_2^\dagger(t)\mathrm{d}_t\mathcal{U}_2(t)$, i.e.,

$$\begin{aligned} \breve{\mathcal{H}} =& \sum_n 2g\cos(\Omega t)\left[\breve{a}_n^\dagger e^{i\omega_n t}\breve{a}_{n+1}e^{-i\omega_{n+1}t}e^{i(\omega_{n+1}-\omega_n)t} + \breve{a}_{n+1}^\dagger e^{i\omega_{n+1}t}\breve{a}_n e^{-i\omega_n t}e^{-i(\omega_{n+1}-\omega_n)t}\right] \\ &+ \sum_n\left(-i\frac{\mathrm{d}}{\mathrm{d}t}(-i\omega_n t)\right)\breve{a}_n^\dagger e^{-i\omega_n t}\breve{a}_n e^{i\omega_n t} \\ =& \sum_n 2g\cos(\Omega t)\left(\breve{a}_n^\dagger\breve{a}_{n+1} + \breve{a}_{n+1}^\dagger\breve{a}_n\right) - \sum_n \omega_n\breve{a}_n^\dagger\breve{a}_n. \quad \text{(effective Hamiltonian 2)} \end{aligned} \tag{12}$$

Similar to the physical interpretation of Eq.(9), Eq.(12) describes the dynamics of a particle with a time-dependent hopping rate and a time-independent on-site potential. Note that $\omega_n = \omega_0 + \omega' n^2$ with $\omega_0 = \gamma H$ and $\omega' = 4\pi^2\gamma A/L^2$ which increases quadratically with site number

n. The negative sign in the on-site potential terms rises from the conventional choice of basis $e^{i\omega t}$ when performing Fourier transformation.

Comparison of results calculated from different Hamiltonian

We have considered different approximations and derived the Hamiltonian $\mathcal{H}$ (Eq.(8) and Eq.(6) in the main text), $\tilde{\mathcal{H}}$ (Eq.(9) and Eq.(7) in the main text) and $\mathcal{H}'$ Eq.(11). Results of two phenomena predicted in the main text, i.e., Bloch oscillation and unidirectional frequency shift, are compared as shown below. The comparison suggests that the choice of gauge does not influence the validity of the model.

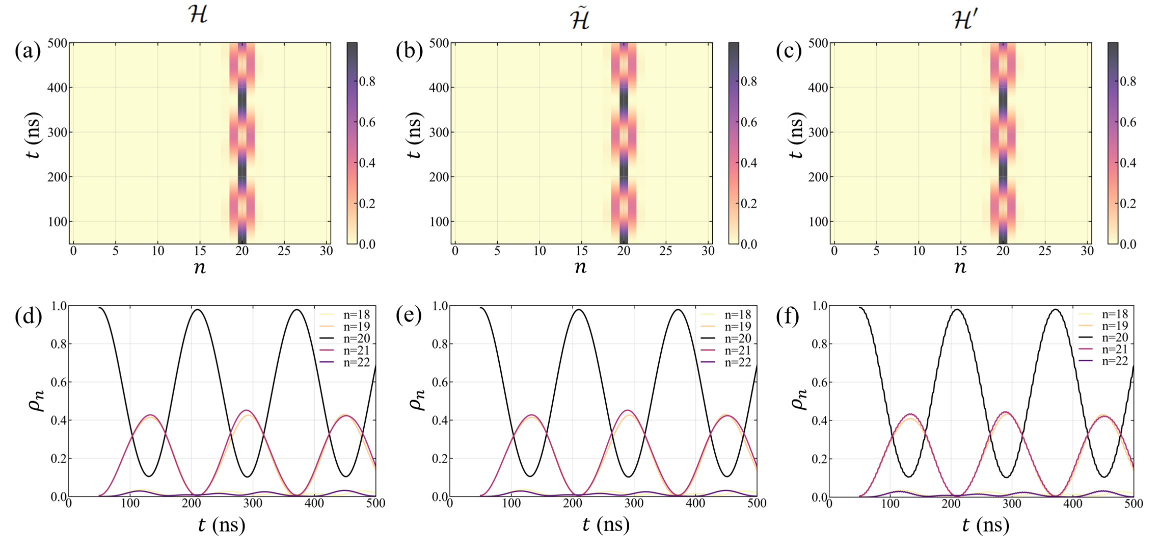

Figure 1: Bloch oscillation calculated from (a)(d) $\mathcal{H}$, (b)(e) $\tilde{\mathcal{H}}$ and (c)(f) $\mathcal{H}'$. Parameters are chosen as $H_\mathrm{m}/H = 0.3\%$ and $\Omega = (\omega_{21} - \omega_{19})/2$, same as Fig.2(b)-(d) in the main text.

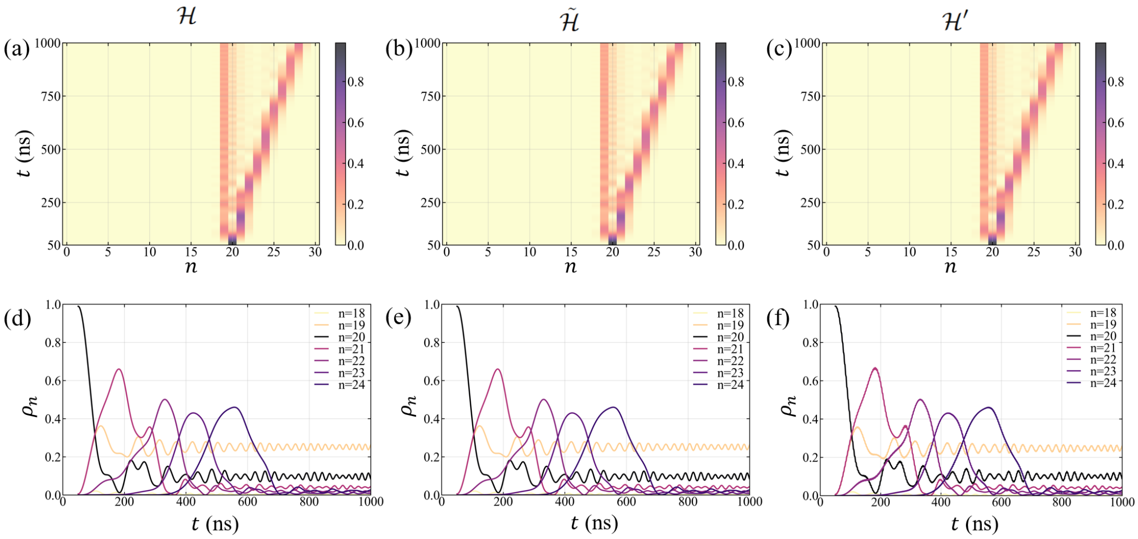

Figure 2: Unidirectional frequency shift calculated from (a)(d) $\mathcal{H}$, (b)(e) $\tilde{\mathcal{H}}$ and (c)(f) $\mathcal{H}'$. Parameters are chosen as $H_\mathrm{m}/H = 0.3\%$, $\Omega_0 = \omega_{20} - \omega_{19}$ and $\kappa = 25\,\mathrm{kHz/ns}$, same as Fig.3(d) in the main text.

Coupling between long-range modes

During the derivation of effective Hamiltonian 1 and 2 (Eq.(9) and Eq.(12)), we assume that the driving frequency is close to the spacing between neighbor modes, i.e., $\Omega \sim \omega_{n+1} - \omega_n$. When ω goes larger, the approximation above is no longer valid and one needs to keep more terms in Eq.(6) to characterize the hopping process which skips multiple sites. For example, when $\Omega \sim \omega_{n+2} - \omega_n$, the effective Hamiltonian including next-nearest neighbor coupling terms reads

$$\begin{aligned}\mathcal{H}'' = \sum_n g \Big[& a_n^\dagger a_{n+1} e^{i(\omega_{n+1}-\omega_n-\Omega)t} + a_{n+1}^\dagger a_n e^{-i(\omega_{n+1}-\omega_n-\Omega)t} \\ & + a_n^\dagger a_{n+2} e^{i(\omega_{n+2}-\omega_n-\Omega)t} + a_{n+2}^\dagger a_n e^{-i(\omega_{n+2}-\omega_n-\Omega)t} \Big] . \end{aligned} \tag{13}$$

Figure 3 shows the numerical results for Bloch oscillation when the next-nearest coupling is included. It is evidenced that the inclusion of next-nearest coupling does not affect the key phenomena (Fig.2 in the main text) as long as the driving frequency is close to the potential difference between neighbour sites. On the other hand, long-range hopping is possible when the driving frequency is matched and the modulation strength is strong enough.

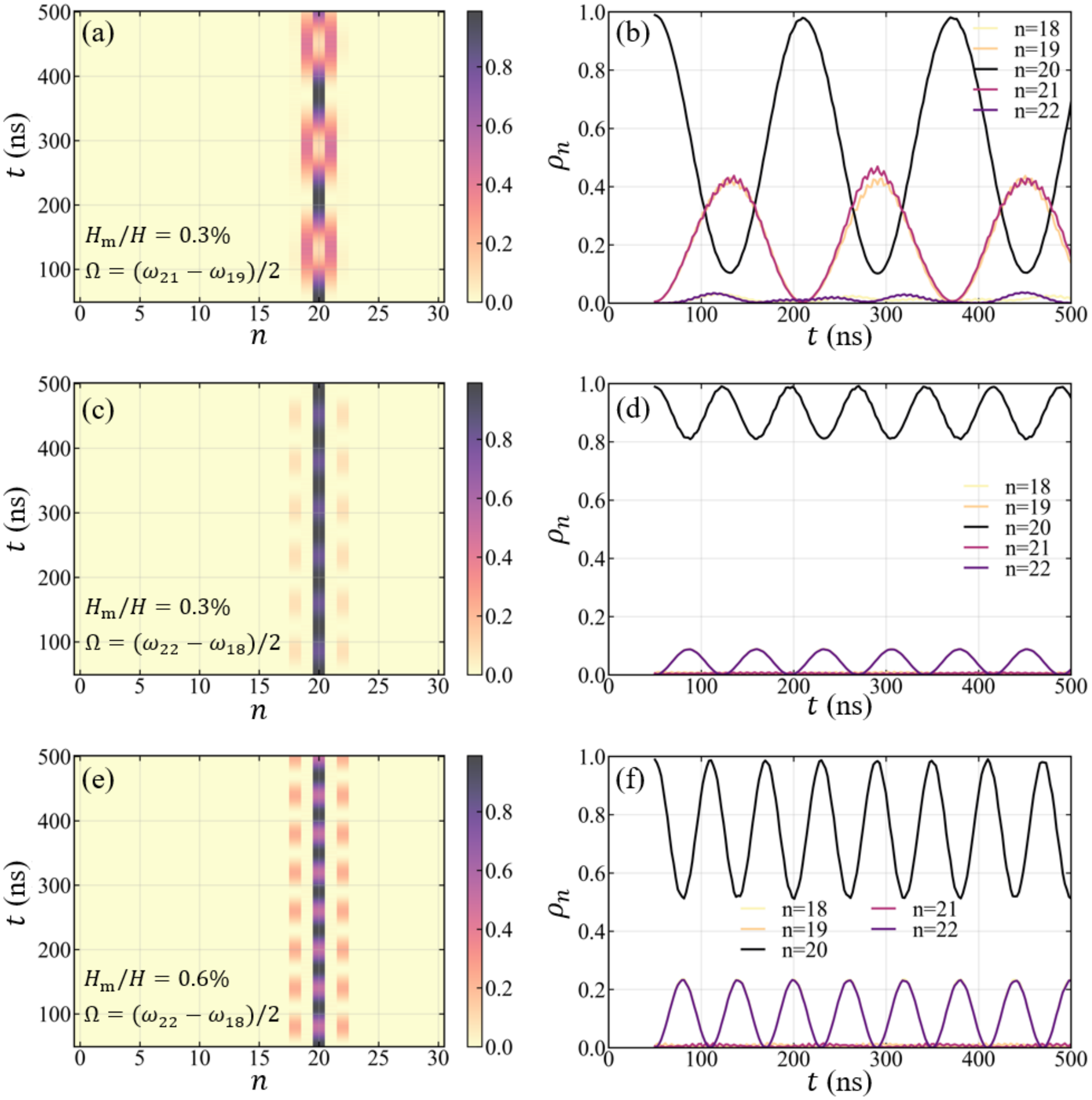

Figure 3: Bloch oscillation calculated from Hamiltonian $\mathcal{H}''$ including next-nearest coupling. (a-b) Frequency of dynamical modulation $\Omega = (\omega_{21} - \omega_{19})/2$ and modulation strength $H_{\rm m}/H = 0.3\%$, working in the same condition as Fig.2 in the main text. (c-d) Frequency of dynamical modulation $\Omega = (\omega_{22} - \omega_{18})/2$ and modulation strength $H_{\rm m}/H = 0.3\%$. (e-f) Frequency of dynamical modulation $\Omega = (\omega_{22} - \omega_{18})/2$ and an enlarged modulation strength $H_{\rm m}/H = 0.6\%$.

Supplementary details for micromagnetic simulations

Temporal evolution produced by micromagnetic simulations using COMSOL

Figure.4(a) shows the temporal evolution and spatial distribution along arc of unit magnetization component m_x. Short-time Fourier transformation is performed on the raw data with 200 windows and overlap factor 0.7, which produces the spectrum shown in Fig. 2(c-d) in the main text.

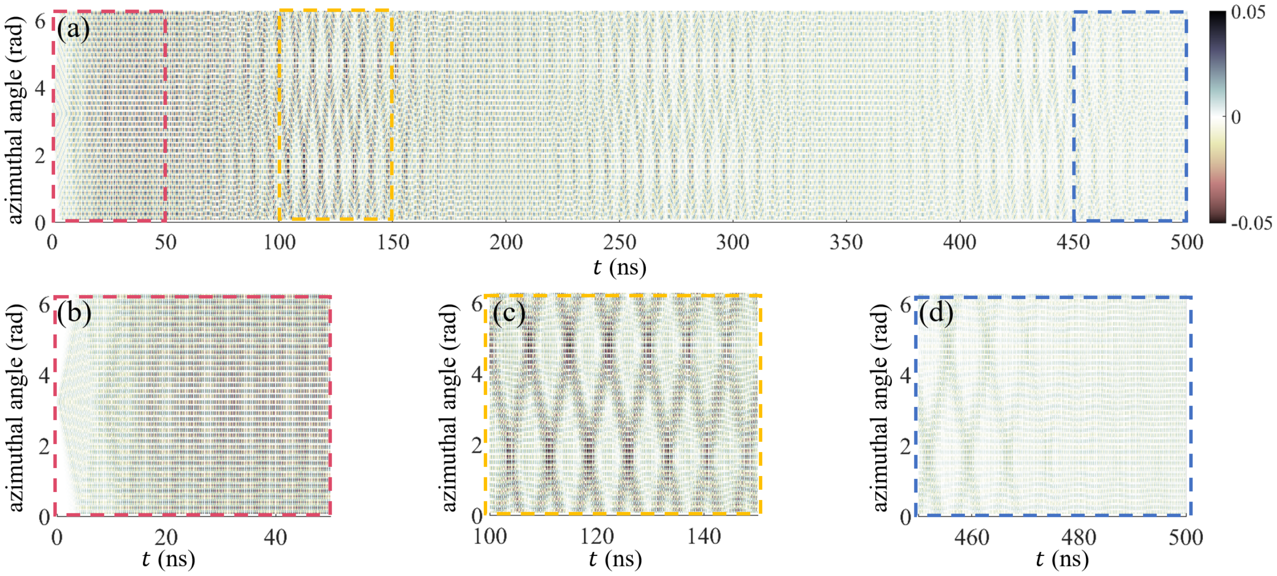

Figure 4: (a) Temporal evolution and spatial distribution of unit magnetization component m_x during the Bloch oscillation scenario (Fig.2 in the main text) obtained from micromagnetic simulation. (b-d) Zoomed-in fragment for different time periods.

Validation of micromagnetic simulations using MuMax3

To validate our COMSOL simulation results, we use the Bloch oscillation case (Fig.2 in the main text) as a benchmark and conduct additional micromagnetic simulations using MuMax3[2].

Figure 5 shows the same conditions as Fig.2(c) without the demagnetizing field, demonstrating perfect agreement with the COMSOL simulation. For cases including the demagnetizing field, we analyze a ring resonator with finite thickness $d = 10\,\mathrm{nm}$ and saturation magnetization $M_s = 0.194 \times 10^6\,\mathrm{A/m}$. We examine two scenarios:

(i) With an external field $H = 0.159 \times 10^5\,\mathrm{A/m}$, comparable to the internal demagnetizing field, Bloch oscillation occurs but with an indeterminate period, as shown in Fig.5(b). This irregularity arises because in this demagnetized-field-dominated regime, the working frequency approaches the spin-wave gap, resulting in more closely spaced energy levels compared to the exchange-dominated regime.

(ii) With a stronger external field $H = 0.339 \times 10^5\,\mathrm{A/m}$, exceeding the demagnetizing field (though its contribution remains significant), the exchange interaction dominates and produces stable Bloch oscillation, as shown in Fig.5(c). However, the higher working frequency induced by the stronger external field leads to faster dynamics and increased damping over the same time period. Consequently, the spin-wave amplitude decays more rapidly than in Fig.5(a).

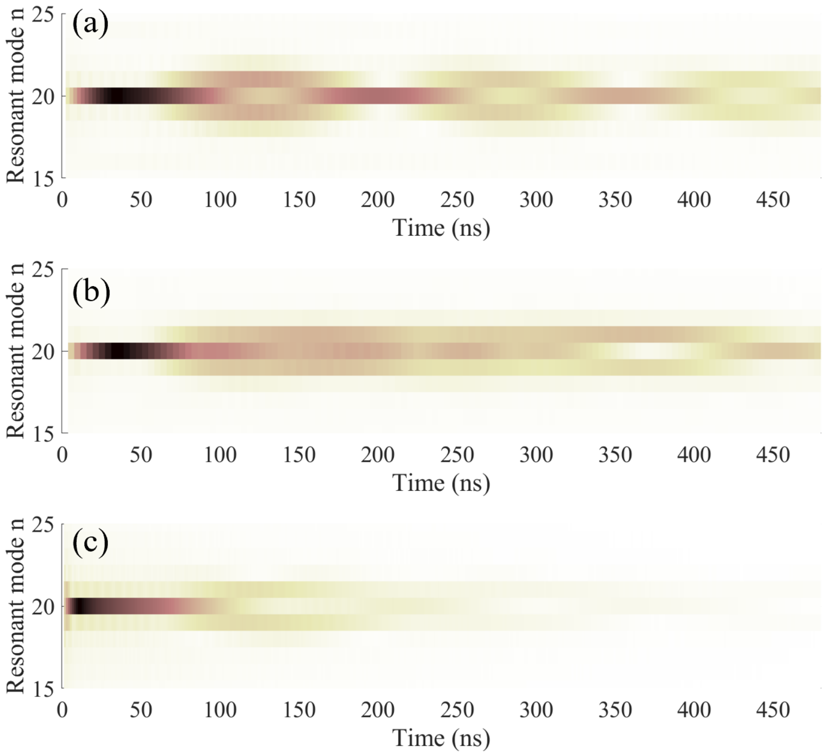

n	*f* (GHz)
21	2.8560
20	2.7235
19	2.5860

n	*f* (GHz)
21	2.5370
20	2.3860
19	2.2350

n	*f* (GHz)
21	8.8810
20	8.7300
19	8.5810

Figure 5: Micromagnetic simulation of Bloch ocillation performed using MuMax3. (a) In the absence of demagnetizing field, all the parameters are consistent with Fig.2(c) in the main text. (b) The demagnetizing field is present with saturation magnetization $M_s = 0.194 \times 10^6\,\mathrm{A/m}$ and external field $H = 0.159 \times 10^5\,\mathrm{A/m}$. (c) The demagnetizing field is present with saturation magnetization $M_s = 0.194 \times 10^6\,\mathrm{A/m}$ and external field $H = 0.339 \times 10^5\,\mathrm{A/m}$. In all cases, the frequency of dynamic modulation is chosen as $\Omega^c_{20} = (\omega_{21} - \omega_{19})/2$.

References

(1) Eckardt, A.; Anisimovas, E. High-frequency approximation for periodically driven quantum systems from a Floquet-space perspective. *New Journal of Physics* **2015**, *17*, 093039.

(2) Vansteenkiste, A.; Leliaert, J.; Dvornik, M.; Helsen, M.; Garcia-Sanchez, F.; Van Waeyenberge, B. The design and verification of MuMax3. *AIP Advances* **2014**, *4*, 107133.
